# Tunable Fano Resonance and Enhanced Sensing in a Simple Au/TiO_2_ Hybrid Metasurface

**DOI:** 10.3390/nano10040687

**Published:** 2020-04-05

**Authors:** Zhihui He, Weiwei Xue, Wei Cui, Chunjiang Li, Zhenxiong Li, Lihui Pu, Jiaojiao Feng, Xintao Xiao, Xuyang Wang, Gang Li

**Affiliations:** School of Physics and Electronic Information, Yan’an University, Yan’an 716000, China; xueww@yau.edu.cn (W.X.); wcui@yau.edu.cn (W.C.); licj@yau.edu.cn (C.L.); lizx@yau.edu.cn (Z.L.); pulh@yau.edu.cn (L.P.); fengjj@yau.edu.cn (J.F.); xiaoxt@yau.edu.cn (X.X.); wangxx@yau.edu.cn (X.W.); ligang0311@csrc.ac.cn (G.L.)

**Keywords:** Fano resonance, plasmonics, sensors

## Abstract

We investigate Fano resonances and sensing enhancements in a simple Au/TiO_2_ hybrid metasurface through the finite-different time-domain (FDTD) simulation and coupled mode theory (CMT) analysis. The results show that the Fano resonance in the proposed simple metasurface is caused by the destructive interaction between the surface plasmon polaritons (SPPs) and the local surface plasmon resonances (LSPRs), the quality factor and dephasing time for the Fano resonance can be effectively tuned by the thickness of Au and TiO_2_ structures, the length of each unit in *x* and *y* directions, as well as the structural defect. In particular, single Fano resonance splits into multiple Fano resonances caused by a stub-shaped defect, and multiple Fano resonances can be tuned by the size and position of the stub-shaped defect. Moreover, we also find that the sensitivity in the Au/TiO_2_ hybrid metasurface with the stub-shaped defect can reach up to 330 nm/RIU and 535 nm/RIU at the Fano resonance 1 and Fano resonance 2, which is more than three times as sensitive in the Au/TiO_2_ hybrid metasurface without the stub-shaped defect, and also higher than that in the TiO_2_ metasurface reported before. These results may provide further understanding of Fano resonances and guidance for designing ultra-high sensitive refractive index sensors.

## 1. Introduction

Surface plasmons (SPs) are collective oscillations of excited electrons at the interface between the metal and dielectric, which can effectively confine and enhance the electric filed [1,2,3,4]. Thus, SPs have important applications in the field of optical sensings, lasers, photovoltaics, and so on [5,6,7,8,9]. Local surface plasmon resonances (LSPRs) is one kind of SPs, which are strongly confined at the surface of metal nanostructures with non-ignorable losses [10,11,12,13,14]. Compared with LSPRs, Surface plasmon polaritons (SPPs) can propagate along the interface with lower losses [15,16,17,18,19]. Many interesting optical phenomena and applications based on LSPRs and SPPs are studied in a variety of nanostructures, such as Fano resonances and plasmon induced transparency (PIT) in nanoparticles or metal-dielectric-metal coupled waveguides [20,21,22,23,24,25,26,27,28,29,30], breaking the optical diffraction limit based on SPs [31,32], extraordinary optical transmission (EOT) in metallic nanohole [33,34,35], enhanced refractive index sensing and slow-light effects in metamaterials and coupled waveguide systems [36,37,38,39,40,41,42,43,44,45].

Fano resonances are caused by the destructive interference between the discrete and continuous states. Different from Lorentz lines, Fano resonances form asymmetric spectral lines, which have very high slope spectra and strong dispersion; the strongly confined electric fields can great enhance the interaction between the light and matters. Thus, Fano resonances are widely used to design ultra-high sensitive sensors in variety of structural systems, such as metamaterials [46,47,48,49], metal-dielectric-metal waveguides [50,51], coupled graphene systems [52,53,54], and so on. Nordlander et al. reported Fano resonances in plasmonic nanostructures and metamaterials [46]. Singh et al. studied the ultra-sensitive terahertz sensing with high-Q Fano resonances in metamaterials [47]. Abujetas et al. investigated Fano resonances and the refractive index sensing in the all dielectric metasurface. They found that the ultra-narrow Fano resonances and the large figure of merit (FOM) due to the minimal loss of the silicon material [48]. Gerislioglu et al. discussed Fano resonances and sensing performances in the metallic metasurface [49]. Lu et al. discussed the plasmonic nanosensor based on Fano resonances in waveguide-coupled resonators [50]. Li et al. studied tunable nanoplasmonic sensors based on the asymmetric degree of Fano resonances in metal-dielectric-metal waveguides [51]. Tang et al. investigated sensing applications based on high-Q Fano resonances in graphene coupled systems [52]. Liu et al. reported high performance of refractive index sensing in the TiO_2_ metasurface generated by the polarization insensitive Fano resonance [55]. Due to the strong optical fields binding characteristics of SPs, the metal and dielectric hybrid metasurface will enhance the sensitivity of the sensor based on Fano resonances. In addition, compared with the metallic metamaterials, narrow spectra of the Fano resonance can be realized in the metal and dielectric hybrid metamaterials. However, there are few studies about Fano resonances and its sensing enhancements in metal and dielectric hybrid metamaterials. Compared with metamaterials, metasurface is a new kind of two-dimensional artificial nanostructure, which is of low loss, easy fabrication, and has advantages in controlling light in nanoscale, so it has important applications in the field of integrated optical devices. Therefore, clarifying the generation and regulation mechanism of the Fano resonance in a simple and easy fabrication hybrid metasurface, and exploring sensing enhancements may play an important role in designing ultra-high sensitivity nano-sensors.

In this paper, we design an Au/TiO_2_ hybrid metasurface and discuss Fano resonances and sensing enhancements in the proposed hybrid metasurface through the coupled mode theory (CMT) and finite-different time-domain (FDTD) simulation. The proposed hybrid metasurface structure is much simpler and easier fabrication than metamaterials. In addition, we find that Fano resonances can be effectively tuned by the structural parameters, the period, and the structural defect. In particular, the multiple Fano resonances can be observed in the Au/TiO_2_ hybrid metasurface with the stub-shaped defect. At last, we investigate the sensing property in the proposed hybrid metasurface, and the result shows that the stub-shaped defect can effectively enhance the sensitivity in the Au/TiO_2_ hybrid metasurface.

## 2. Structure and Theory Model

**Figure 1:**
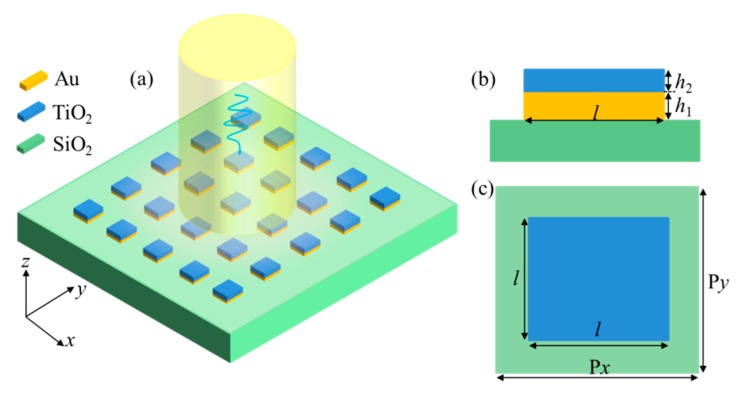
(**a**) schematic diagram of the Au/TiO_2_ hybrid metasurface; (**b**) *x*–*z* plane view of the proposed hybrid metasurface; (**c**) *x*–*y* plane view of the proposed hybrid metasurface.

As shown in Figure 1a, we propose a metal and dielectric hybrid metasurface. The metal and dielectric are chosen to be Au and TiO_2_, respectively, and the substrate is glass, the permittivity of Au, TiO_2_, and glass reference to the related articles [35,55]. Figure 1b,c show the *x*–*z* plane view and *x*–*y* plane view of the proposed hybrid metasurface. *l =* 200 nm is the length of Au and TiO_2_ structures, and *h*_1_ and *h*_2_ are the thickness of Au and TiO_2_ structures, respectively. P*x* and P*y* are the length of each cell in the *x-* and *y*-directions, respectively. The transmission spectra of the proposed Au and TiO_2_ hybrid metasurface are simulated by use of the FDTD simulation method. In this simulation, the effective area is divided into uniform Yee cells with the spatial step Δ*x* = Δ*y* = Δ*z* = 1 nm and the time step Δ*t* = Δ*x*/2*c* (*c* is the velocity of light in vacuum) [6], and the perfectly matched layer (PML) is chosen in the *z*-direction, and the periodic boundary condition is set in *x-* and *y*-directions [6,35]. The Gaussian beam with the *x*-directional polarization incidents from the positive direction of a *z*-axis in our simulation. 

**Figure 2:**
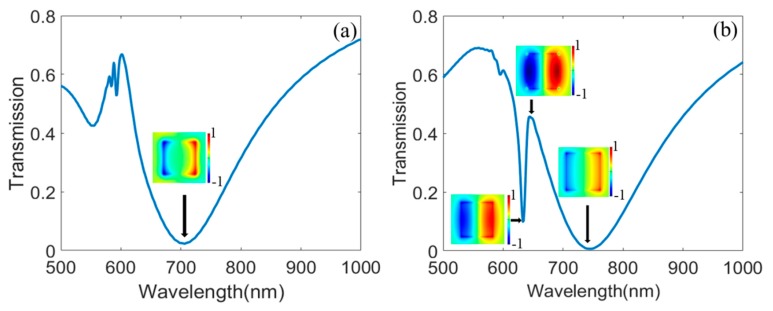
(**a**) transmission spectrum of the metasurface when there is only the Au structure on the substrate with *h*_1_ = 90 nm, *l =* 200 nm, and P*x* = P*y* = 400 nm, the inset figure is the electric field distribution E*z* at the wavelength of 705 nm, (**b**) transmission spectrum of the proposed Au/TiO_2_ hybrid metasurface with *h_1_ =* 90 nm, *h_2_ = 50* nm, *l =* 200 nm, and P*x* = P*y* = 400 nm, the insets are the electric field distributions E*z* at the wavelength of 632.1 nm, 653.4 nm, and 737.6 nm.

The transmission spectra of the proposed Au/TiO_2_ hybrid metasurface are plotted in Figure 2. Figure 2a shows the transmission spectrum of the metasurface when there is only the Au structure on the substrate with *h*_1_ = 90 nm, *l =* 200 nm, and P*x* = P*y* = 400 nm. We can see that a transmission dip appears at the wavelength of 705 nm. The inset of the Figure 2a depicts the electric field distribution E*z* at the wavelength of 705 nm on the surface of the Au structure. We can see that the transmission dip is caused by the LSPRs at the boundary of the Au structure. Then, we investigate the transmission spectrum of the proposed Au/TiO_2_ hybrid metasurface with *h*_1_ = 90 nm, *h*_2_ = 50 nm, *l =* 200 nm, and P*x* = P*y* = 400 nm. We can see that the typical Fano resonance spectrum can be observed in Figure 2b. The electric field distribution E*z* on the interface between the Au and TiO_2_ structure at the transmission peak and dips are shown in insets of Figure 2b. From the electric field distribution E*z*, we can see that SPPs are excited at the interface between Au and TiO_2_ structures, and the destructive interaction between the LSPRs and SPPs causes the Fano resonance in the proposed hybrid metasurface.

**Figure 3:**
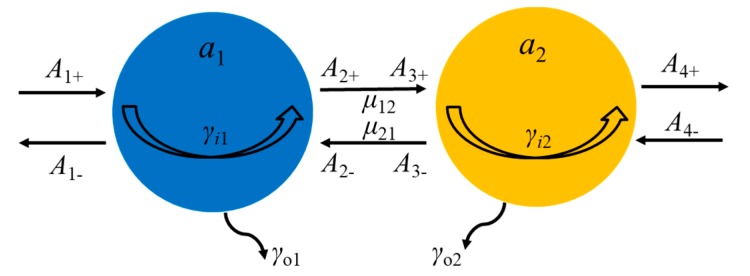
Schematic diagram of coupled mode theory (CMT) for the proposed Au/TiO_2_ hybrid metasurface.

In order to further understand Fano resonances in the proposed metasurface, we introduce CMT to theoretically analyze the generation and regulation mechanisms of Fano resonances. Based on coupling characteristics of resonant modes, we plot the schematic diagram of CMT for the proposed Au/TiO_2_ hybrid metasurface as shown in Figure 3.

Here, *a*_1_ and *a*_2_ are defined as complex amplitudes of LSPRs and SPPs. *ω* is the angular frequency of the incident wave, and *ω*_1_ and *ω*_2_ are resonant angular frequencies of LSPRs and SPPs, respectively. *γ_i_*_1(2)_ = *ω*_1(2)_/(2*Q_i_*_1(2)_) stands for the decay of LSPRs and SPPs due to the intrinsic loss, *Q_i_*_1(2)_ are the quality factor of LSPRs and SPPs modes. *γ_o_*_1(2)_ = *ω*_1(2)_/(2*Q_o_*_1(2)_) are the decays due to the energy escaping into the air, *Q_o_*_1(2)_ are quality factors of coupling. *μ*_12_ = *μ*_21_ is the direct coupling between LSPRs and SPPs in the proposed hybrid metasurface. *A*_N±_ (N = 1, 2, 3, and 4) represent the amplitudes of the input and output waves. Thus, the CMT equation for our proposed hybrid metasurface can be expressed as follows [6,23]:

(1) $$-j\omega a_{1}=\left(-j\omega_{1}-\gamma_{i1}-\gamma_{o1}\right)a_{1}+A_{1+}\sqrt{\gamma_{o1}}+A_{2-}\sqrt{\gamma_{o1}}+j\mu_{12}a_{2},$$

(2) $$-j\omega a_{2}=\left(-j\omega_{2}-\gamma_{i2}-\gamma_{o2}\right)a_{2}+A_{3+}\sqrt{\gamma_{o2}}+A_{4-}\sqrt{\gamma_{o2}}+j\mu_{21}a_{1}.$$

In order to solve the transmission coefficient of the proposed system, we assume *ζ*_1_ and *ζ*_2_ as:

(3) $$\zeta_{1}=j\omega -j\omega_{1}-\gamma_{i1}-\gamma_{o1},\;\zeta_{2}=j\omega -j\omega_{2}-\gamma_{i2}-\gamma_{o2}.$$

Thus, Equations (1) and (2) can be simplified as:

(4) $$\zeta_{1}a_{1}+A_{1+}\sqrt{\gamma_{o1}}+A_{2-}\sqrt{\gamma_{o1}}+j\mu_{12}a_{2}=0,$$

(5) $$\zeta_{2}a_{2}+A_{3+}\sqrt{\gamma_{o2}}+A_{4-}\sqrt{\gamma_{o2}}+j\mu_{21}a_{1}=0.$$

Here, we assume that the light wave is lossless as it propagates through the space, so the law of energy conservation can be performed. Thus, the relationship among the *A*_N±_ can be expressed as follows:

(6) $$A_{2+}=A_{1+}-a_{1}\sqrt{\gamma_{o1}},\;A_{4+}=A_{3+}-a_{2}\sqrt{\gamma_{o2}},$$

(7) $$A_{1-}=A_{2-}-a_{1}\sqrt{\gamma_{o1}},\;A_{3-}=A_{4-}-a_{2}\sqrt{\gamma_{o2}},$$

(8) $$A_{3+}=A_{2+}\exp\left(j\varphi \right),\;A_{3-}=A_{2-}\exp\left(-j\varphi \right),$$

where *φ* is the indirect coupling phase between LSPRs and SPPs. In our proposed coupled mode system, we assume that *A*_4_ equals to 0 in the proposed structure. Then, we apply Equations (6)–(8) to Equations (4) and (5). The transmission coefficient in the proposed hybrid metasurface *t* = *A*_4+_/*A*_1+,_ Thus, the transmittance *T* = │*A*_4+_/*A*_1+_│^2^ can be expressed as:

(9) $$T={\left|\exp\left(j\varphi \right)+\frac{\gamma_{i1}\zeta_{2}\exp\left(j\varphi \right)+\gamma_{i2}\zeta_{1}\exp\left(j\varphi \right)+\sqrt{\gamma_{i1}\gamma_{i2}}\exp\left(2j\varphi \right)\chi_{1}+\sqrt{\gamma_{i1}\gamma_{i2}}\chi_{2}}{\zeta_{1}\zeta_{2}-\chi_{1}\chi_{2}}\right|}^{2},$$

with

(10) $$\chi_{1}=\sqrt{\gamma_{i1}\gamma_{i2}}\exp\left(j\varphi \right)+j\mu_{12},\;\chi_{2}=\sqrt{\gamma_{i1}\gamma_{i2}}\exp\left(j\varphi \right)+j\mu_{21}.$$

## 3. Results and Discussion

### 3.1. Tunable Fano Resonances in the Hybrid Metasurface

**Figure 4:**
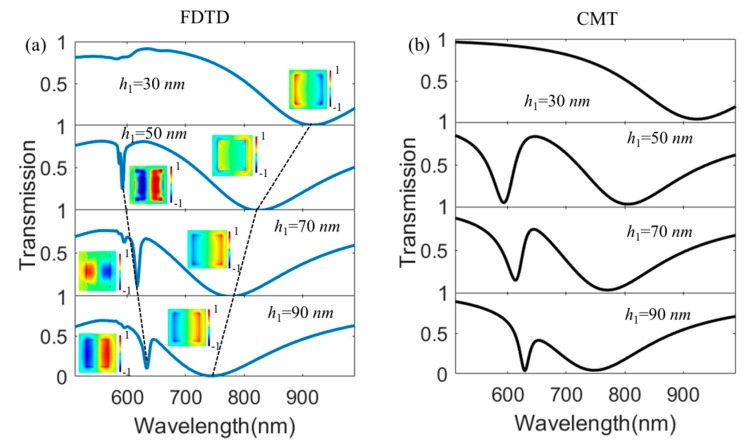
(**a**) FDTD simulation results of transmission spectra as a function of the thickness of *h*_1_ for the proposed hybrid metasurface when *h*_2_ = 50 nm, *l =* 200 nm, and Px = Py = 400 nm, the inset is the electric field distribution E*z* at the transmission dips; (**b**) CMT results of transmission spectra as a function of the thickness of *h*_1_ = 90 nm (*φ* = 0.48 π, *Q_i_*_1_ = 58, *Q_i_*_2_ = 406, *Q_o_*_1_ = 291, *Q_o_*_2_ = 300, and *μ*_12_ = *μ*_21_ = 5.1 × 10^13^), *h*_1_ = 70 nm (*φ* = 0.48 π, *Q_i_*_1_ = 58, *Q_i_*_2_ = 387, *Q_o_*_1_ = 296, *Q_o_*_2_ = 305, and *μ*_12_ = *μ*_21_ = 7.9 × 10^13^), *h*_1_ = 50 nm (*φ* = 0.48 π, *Q_i_*_1_ = 58, *Q_i_*_2_ = 356, *Q_o_*_1_ = 294, *Q_o_*_2_ = 301, and *μ*_12_ = *μ*_21_ = 9.2 × 10^13^), and *h*_1_ = 30 nm (*φ* = 0.48 π, *Q_i_*_1_ = 58, *Q_i_*_2_ = 293, *Q_o_*_1_ = 289, *Q_o_*_2_ = 295, and *μ*_12_ = *μ*_21_ = 15.4 × 10^13^).

Firstly, we investigate the dependence of Fano resonance on the thickness of the Au nanostructure *h*_1_ in our proposed hybrid metasurface when *h*_2_ = 50 nm, *l =* 200 nm, and P*x* = P*y* = 400 nm. Figure 4a shows transmission spectra as *h*_1_ increases from 30 nm to 90 nm by use of the FDTD simulation method. The results show that there is no Fano resonance phenomenon when *h*_1_ = 30 nm; this is because there is strong coupling between LSPRs and SPPs, the electric filed distribution E*z* at the transmission dip as shown in the inset can explain this phenomenon as well. With the increasing of *h*_1_, the direct coupling between LSPRs and SPPs decreases, and resonant wavelengths of LSPRs and SPPs show blue and red shift, respectively. Thus, the Fano resonance becomes more and more obvious as shown in Figure 4a. Then, we introduce the CMT to describe transmission spectra versus the thickness of the Au nanostructure as depicted in Figure 4b. From Figure 4a,b, we can see that FDTD simulation results are in agreement with CMT results.

**Figure 5:**
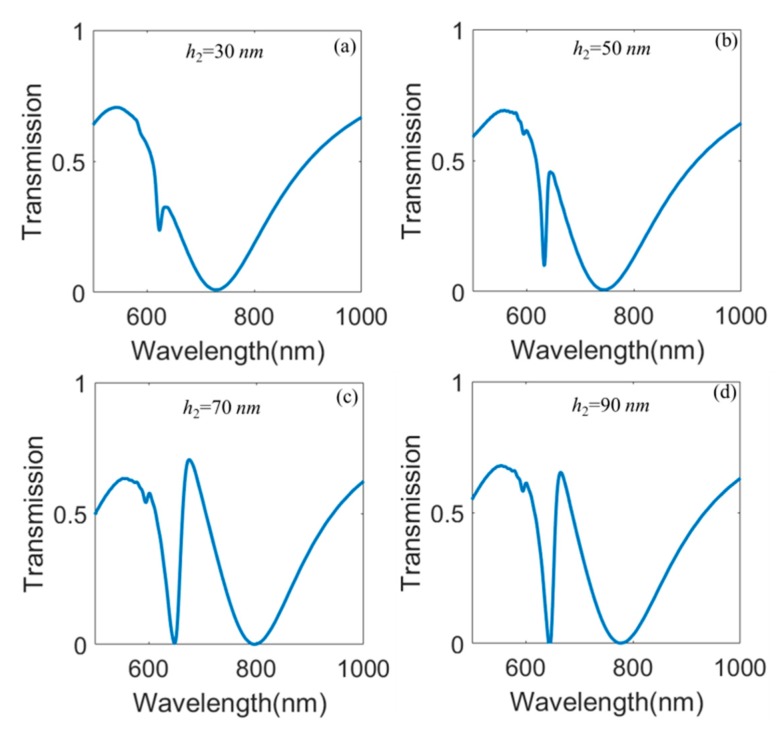
Transmission spectra as a function of the thickness of *h*_2_ in the proposed hybrid metasurface when *h*_1_ = 90 nm, *l =* 200 nm, and P*x* = P*y* = 400 nm.

Then, we will discuss transmission spectra as a function of the thickness of TiO_2_ layer as depicted in Figure 5. From Figure 5a, we can see that there are two transmission dips when *h*_2_ = 30 nm. The left transmission dip is caused by SPPs on the interface between Au and TiO_2_ layers, and the right transmission dip is the result of LSPRs. We can also see that the transmission ratio of the left transmission dip is very large as shown in Figure 5a because the thin TiO_2_ layer can not effectively confine SPPs on the interface of Au and TiO_2_ layers. As the thickness of TiO_2_ layer *h*_2_ increases, the transmission ratio of the left transmission dip decreases to zero as described in Figure 5b–d. In particular, the Fano resonance becomes more and more obvious when *h*_2_ increases from 30 nm to 90 nm.

Here, we introduce the definition of quality factor *Q*_F_ = *δ*/*ω*_F_ − *ω*_F_/4*δ* to discuss the Fano resonance in our proposed hybrid metasurface [56,57], where *δ =* │*ω*_D1_ − *ω*_D2_│, *ω*_D1_, *ω*_D2_, *ω*_F_ are resonant frequencies at the transmission dips and peak for the induced Fano resonance, respectively. In addition, the dephasing time of the Fano resonance is a critical parameter that can be defined by taking into account the resonant narrowness as follows [57]: *t_d_* = 2*ħδ*. The quality factor *Q*_F_ and dephasing time *t_d_* of the Fano resonance in our proposed Au/TiO_2_ hybrid metasurface as functions of the thichness *h*_1_ and *h*_2_ are shown in Table 1. From Table 1, we can see that the quality factor *Q*_F_ and dephasing time *t_d_* increases when the thickness of the Au layer *h*_1_ increases. For the factor *h*_2_, we can see that the quality factor *Q*_F_ and dephasing time *t_d_* do not change monotonically. However, the maximum of the *Q*_F_ = 15.3 and *t_d_* = 28.1 *fs* can be observed in our proposed hybrid metasurface when *h*_1_ = 90 nm and *h*_2_ = 50 nm. This result will provide the guidance for tuning Fano resonance spectra and the dephasing time *t_d_* in the metal and dielectric hybrid metasurface.

**Figure 6:**
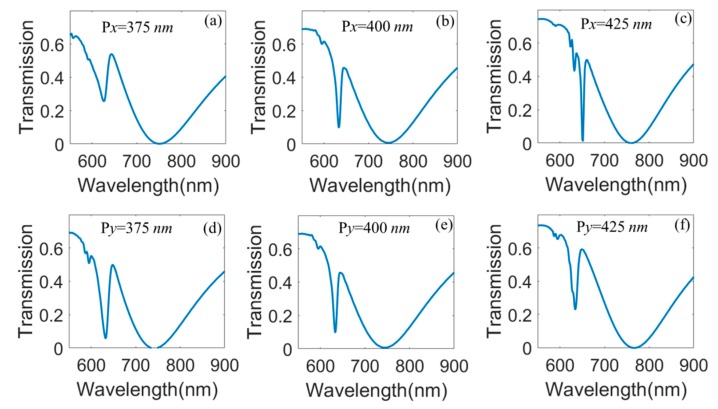
(**a**–**c**) transmission spectra as a function of P*x* for the proposed hybrid metasurface when *h*_1_ = 90 nm, *h*_2_ = 50 nm, *l =* 200 nm, and Py = 400 nm; (**d**–**f**) transmission spectra as a function of Py when *h*_1_ = 90 nm, *h*_2_ = 50 nm, *l =* 200 nm, and P*x* = 400 nm.

Generally speaking, the length of each unit in *x-* and *y*-directions has great influence on transmission spectra, so we will study the effect of P*x* and P*y* on Fano resonances in our proposed hybrid metasurface as shown in Figure 6. Figure 6a–c show transmission spectra as a function of P*x* when *h*_1_ = 90 nm, *h*_2_ = 50 nm, *l =* 200 nm, and P*y* = 400 nm. We can see that the transmission ratio of the left transmission dip obviously decreases, and the transmission spectra show slight red shift as P*x* increases. This phenomenon is caused by the coupling between the adjacent structural unit decreases with the increasing of P*x*. Then, we discuss the transmission spectra as a function of P*y* when *h*_1_ = 90 nm, *h*_2_ = 50 nm, *l =* 200 nm, and P*x* = 400 nm as depicted in Figure 6d–f. We can see that the transmission spectra also show red shift. However, the transmission ratio of the left transmission dip increases when P*y* increases, which shows the opposite effect compared with Figure 6a–c. This is because that the *x*-directional polarization of light is chosen in our FDTD simulation. We also calculate the quality factor *Q*_F_ and dephasing time *t_d_* of the Fano resonance when the period P*x* and P*y* equal to 375 nm, 400 nm, and 425 nm in the proposed hybrid metasurface. We find that the effect of P*x* and P*y* on the quality factor *Q*_F_ and dephasing time *t_d_* is much less than that for the thickness *h_1_* and *h_2_*.

**Figure 7:**
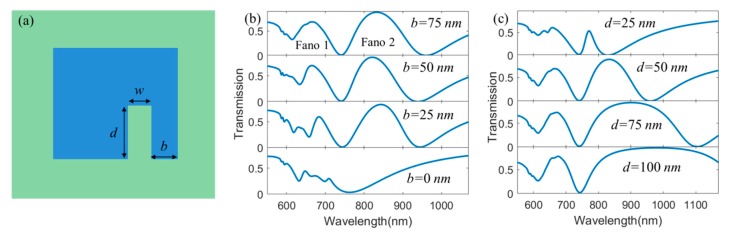
(**a**) *x*–*y* plane view of the schematic diagram for the Au/TiO_2_ metasurface with the stub-shaped defect, (**b**) transmission spectra as a function of *b* when *d =* 50 nm, *w =* 50 nm, *h_1_ =* 90 nm, *h_2_ =* 50 nm, *l =* 200 nm, *and* P*x =* P*y =* 400 nm; (**c**) transmission spectra as a function of *d* when *b* = 75 nm, *w* = 50 nm, *h_1_ =* 90 nm, *h_2_ =* 50 nm, *l =* 200 nm, and P*x* = P*y* = 400 nm.

Finally, we investigate the effect of the structural defect on Fano resonances in our proposed hybrid metasurface. Here, we introduce a stub-shaped defect in our proposed structure as shown in Figure 7a, *w* is the width of the stub, *d* is the height of the stub, and *b* is the distance between the stub and the right side of the structure. Figure 7b describes transmission spectra as a function of *b* when *d* = 50 nm, *w* = 50 nm, *h*_1_ = 90 nm, *h*_2_ = 50 nm, *l =* 200 nm, and P*x* = P*y* = 400 nm. The results show that the resonant wavelength of LSPRs increases when *b* increases from 0 to 75 nm. Furthermore, interesting, single Fano resonance splits into multiple Fano resonances when *b* increases, which is caused by the present of additional cavity resonance mode besides LSPRs and SPPs in the hybrid metasurface. Then, we study the effect of *d* on transmission spectra when *b* = 75 nm, *w* = 50 nm, *h*_1_ = 90 nm, *h*_2_ = 50 nm, *l =* 200 nm, and P*x* = P*y* = 400 nm as shown in Figure 7b. We can also see that the resonant wavelength of LSPRs increases and obvious double Fano resonances appear when *d* increases from 25 nm to 75 nm. These results may provide important guidance for regulation of Fano resonances in the Au/TiO_2_ hybrid metasurface.

Finally, we also discuss the quality factor *Q*_F_ and dephasing time *t_d_* of the Fano resonance in the hybrid metasurface with a stub-shaped defect as shown in Table 2. Fano 1 is the left Fano resonance, and Fano resonance 2 stands for the right Fano resonance in Figure 7b,c. Observing from Table 2, we can see that the quality factors *Q*_F_ for the Fano resonance 1 and 2 decrease as the length *d* increases. Meanwhile, the dephasing time *t_d_* also decreases with the increasing of *d*. The maximum of dephasing time *t_d_* can reach up to 46.4 *fs* at the Fano resonance 2 when *d* = 25 nm, *b* = 75 nm, *w* = 50 nm, *h*_1_ = 90 nm, *h*_2_ = 50 nm, *l =* 200 nm, and P*x* = P*y* = 400 nm. Moreover, the quality factor *Q*_F_ and dephasing time *t_d_* for Fano resonance 1 show obvious increase when *b* increases. However, the quality factor *Q*_F_ and dephasing time *t_d_* for the Fano resonance 2 shows the moderate trend as *b* increases.

### 3.2. Sensing Enhancement Based on the Stub-Shaped Defect

**Figure 8:**
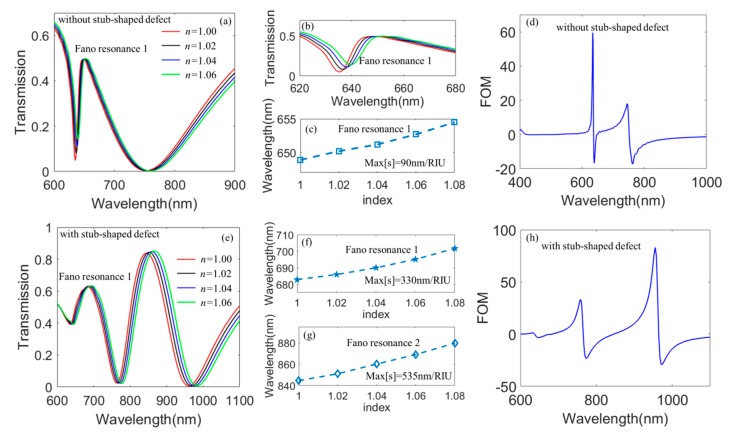
(**a**) transmission spectra as a function of *n* when *h_1_ =* 90 nm, *h_2_ =* 50 nm, *l =* 200 nm, and P*x* = P*y* = 400 nm in the Au/TiO_2_ hybrid metasurface without the stub-shaped defect; (**b**) magnification of the Fano resonance in Figure 8a; (**c**) FOM in the proposed hybrid metasurface without stub-shaped defect; (**d**) resonant wavelength of Fano resonance versus *n* in Figure 8b; (**e**) transmission spectra as a function of the refractive index for the external environment when *b* = 75 nm, *d* = 75 nm, *w* = 50 nm, *h_1_ =* 90 nm, *h_2_ =* 50 nm, *l =* 200 nm, and P*x* = P*y* = 400 nm in the Au/TiO_2_ metasurface with the stub-shaped defect; (**f**) resonant wavelength of the Fano resonance 1 versus *n* in Figure 8d; (**g**) resonant wavelength of the Fano resonance 2 versus *n* in Figure 8d; (**h**) FOM in the proposed hybrid metasurface with a stub-shaped defect.

As is well known, the steep spectra of the Fano resonance can effectively enhance the sensitivity of refractive index sensing. Here, we further study the mechanism of the sensing enhancement by the defect in the proposed hybrid metasurface as shown in Figure 8. We investigate the transmission spectra versus the refractive index of the external environment when *h*_1_ = 90 nm, *h*_2_ = 50 nm, *l =* 200 nm, and P*x* = P*y* = 400 nm in the Au/TiO_2_ hybrid metasurface when there are no stub-shaped defects. We can see that the Fano resonance shows a red shift when the refractive index *n* increases from 1.00 to 1.06. Figure 8c shows the resonant wavelength of the Fano resonance peak versus *n*; we can find that the resonant wavelength shows a linear increase as the refractive index *n* increases. The sensitivity is the most important physical parameter to measure sensor characteristics of the structure. Here, we introduce the definition of the sensitivity as *s* = Δ*λ*/Δ*n*, where *λ* is the resonant wavelength of the Fano resonance. According to this equation, we can calculate the maximum of the sensitivity Max(*s*)= 90 nm/*RIU* in the proposed hybrid metasurface without the stub-shaped defect. Figure 8e plots the transmission spectra as a function of the refractive index for the external environment when *b* = 75 nm, *d* = 50 nm, *w* = 50 nm, *h*_1_ = 90 nm, *h*_2_ = 50 nm, *l =* 200 nm, and P*x* = P*y* = 400 nm in the Au/TiO_2_ hybrid metasurface with the stub-shaped defect. We can see that the transmission spectra show obvious red shift as the refractive index *n* increases. The resonant wavelength of the Fano resonance 1 and 2 versus *n* are depicted in Figure 8f,g. We can see that the resonant wavelengths of the Fano resonance 1 and 2 increase with the increasing of *n*. In addition, we calculate the maximum of sensitivity at the Fano resonance 1 and 2, and Max(*s*) = 330 nm/RIU for the Fano resonance 1, Max(*s*) = 535 nm/RIU for the Fano resonance 2. In particular, the sensitivity in the hybrid metasurface with a stub-shaped defect is three times larger than that in Figure 8c. More importantly, the sensitivity in the proposed Au/TiO_2_ hybrid metasurface is much higher than that in the pure TiO_2_ metasurface reported in the recent reference [53]. Finally, we investigate the figure of merit (FOM) in our proposed hybrid metasurface as shown in Figure 8d,h. FOM is defined as (ΔT/Δn)/T  [58], where ∆(*T*)/∆(*n*) is the relative intensity change induced by the refractive index change ∆*n*. Figure 8d shows the FOM of sensing at the fixed wavelength when there is no defect on the proposed Au/TiO_2_ hybrid metasurface. We can see that the maximum of FOM can reach up to 60 at the wavelength of 634.1 nm. The FOM for the proposed Au/TiO_2_ hybrid metasurface with a stub-shaped defect are shown in Figure 8h. In addition, the maximum of the FOM are equal to 91 at the wavelength of 971 nm when *b* = 75 nm, *d* = 50 nm, *w* = 50 nm, *h*_1_ = 90 nm, *h*_2_ = 50 nm, *l =* 200 nm, and P*x* = P*y* = 400 nm. These results will have great significance for designing ultra-high sensitive nanosensors.

## 4. Conclusions

In summary, we have studied Fano resonances and its sensing enhancements in the simple Au/TiO_2_ metasurface by use of the FDTD simulation method and CMT analysis. We find that the Fano resonance in the proposed hybrid metasurface is caused by the destructive interaction between SPPs and LSPRs, and the line shape, quality factor *Q*_F_, and dephasing time *t_d_* of the Fano resonance can be effectively tuned by the thickness of the Au layer and TiO_2_ layer, the length of each period in the *x-* and *y-*directions as well as the structure defect. It is more interesting to note that the stub-shaped defect can make a single Fano resonance spectrum split into multiple Fano resonances, and multiple Fano resonances can be tuned by the size and position of the stub-shaped defect. Moreover, the sensitivity and the FOM in the proposed hybrid metasurface have been studied in detail in our work. We find that the refractive index sensitivity in the Au/TiO_2_ hybrid metasurface with the stub-shaped defect can research up to 330 nm/RIU and 535 nm/RIU at the Fano resonance 1 and Fano resonance 2, which is much larger than that in the Au/TiO_2_ hybrid metasurface without the stub-shaped defect, and the sensor performance is also obviously better than that in a pure TiO_2_ metasurface. We also find that the maximum of the FOM can reach up to 91 at the wavelength of 971 nm when the proposed hybrid metasurface contains the stub-shaped defect. These results may provide a deep understanding of Fano resonances and guidance for designing higher sensitive refractive index sensors.

## Figures and Tables

**Table 1:** Quality factor *Q*_F_ and dephasing time *t_d_* versus *h*_1_ and *h*_2._

	*h*_1_ (nm)				*h*_2_ (nm)			
	30	50	70	90	30	50	70	90
***Q*_F_**	0.4	4.6	8.5	15.3	14.0	15.3	10.2	12.5
***t_d_* (fs)**	10.7	14.0	19.9	28.1	27.3	28.1	23.7	25.0

**Table 2:** Quality factor *Q*_F_ and dephasing time *t_d_* versus *d* and *b*.

		*d* (nm)				*b* (nm)		
		25	50	75	100	25	50	75
***Q*_F_**	Fano 1	12.4	9.3	9.3	8.9	9.1	11.1	16.3
	Fano 2	23.4	6.8	3.6	2.4	6.7	7.7	7.7
***t_d_* (fs)**	Fano 1	32.7	24.4	24.3	23.5	24.0	29.5	38.5
	Fano 2	46.4	21.5	15.0	12.1	20.2	23.1	23.2
